# Phase 1/1b open-label, dose-escalation study of fruquintinib in patients with advanced solid tumors in the United States

**DOI:** 10.1007/s10637-023-01395-y

**Published:** 2023-10-05

**Authors:** Andrea Wang-Gillam, William Schelman, Stacey Ukrainskyj, Caly Chien, Martha Gonzalez, Zhao Yang, Marek Kania, Heather Yeckes-Rodin

**Affiliations:** 1 Washington University School of Medicine, St. Louis, MO USA; 2 HUTCHMED International Corporation, Florham Park, NJ USA; 3 Hematology Oncology Associates of the Treasure Coast, Port St. Lucie, FL USA

**Keywords:** Angiogenesis, Fruquintinib, Solid tumors, Tyrosine kinase inhibitor, Vascular endothelial growth factor receptors

## Abstract

This open-label, phase 1/1b study was conducted to evaluate the safety, tolerability, and pharmacokinetics (PK) of fruquintinib in United States (U.S.) patients to confirm the recommended phase 2 dose (RP2D) established in China. Patients with advanced solid tumors who had progressed on approved systemic therapy, were enrolled into 2 successive dose escalation cohorts, fruquintinib 3 mg (n = 7) or 5 mg (n = 7), orally, once daily (QD), 3 weeks on and 1 week off (3/1) with a 3 + 3 design followed by a dose expansion cohort at the RP2D 5 mg dose (n = 6). PK samples were collected on Days 1, 14, and 21 (Cycle 1). One of 6 dose-limiting toxicity (DLT)-evaluable patients in the 3 mg cohort had a DLT of grade 4 hypertension; there were no DLTs in the 5 mg cohort. The RP2D was confirmed to be 5 mg QD 3/1. All 20 patients experienced a treatment-emergent adverse event; grade ≥ 3 in 5 (71.4%; 3 mg dose) and 12 (92.3%; 5 mg dose) patients. Two patients had a confirmed partial response. After single and multiple doses, median peak plasma concentrations occurred at 2 h post-dose. Steady-state was achieved after 14 days of QD dosing with systemic exposure four-fold higher than that after a single dose. Fruquintinib was well tolerated, and the safety and PK profile at the 5 mg RP2D in U.S. patients with advanced solid tumors was consistent with dose-finding studies in China. Preliminary anticancer activity was observed. This study is registered at *Clinicaltrials.gov NCT03251378*.

**Supplementary Information:**

The online version contains supplementary material available at 10.1007/s10637-023-01395-y.

## Introduction

Fruquintinib (HMPL-013) is a highly selective, potent, oral, small-molecule tyrosine kinase inhibitor of vascular endothelial growth factor receptors (VEGFR) -1, -2, and -3 [1, 2]. Notably, in preclinical studies, fruquintinib demonstrated improved kinase selectivity over currently approved VEGFR inhibitors [1]. The optimal fruquintinib dose schedule was determined in 2 phase 1 studies conducted in China. The first study (2009–013-00CH1; NCT01645215) investigated doses of fruquintinib at 1, 2, 4, 5, and 6 mg administered once daily (QD) continuously, and then 5 and 6 mg QD administered with an intermittent dosing schedule of 3 weeks on and 1 week off (QD 3/1) in 28-day cycles [3]. The maximum-tolerated dose (MTD)/recommended phase 2 dose (RP2D) was 4 mg QD continuous or 5 mg QD 3/1. In the second study (2012–013-00CH3; NCT01975077), the safety and tolerability of these 2 dosing regimens were compared in patients with metastatic colorectal cancer (mCRC) [4]. The safety profile was better in the 5 mg QD 3/1 cohort than in the 4 mg QD continuous cohort [4]. Thus, the 5 mg QD 3/1 regimen was selected as the RP2D and was further tested in a randomized, double-blind, placebo-controlled phase 2 study (2012–013-00CH1; NCT02196688). This phase 2 study confirmed that the dosing regimen of 5 mg QD 3/1 was safe and effective in patients with refractory mCRC and established the standard dose and dosing regimen in all other studies in patients with advanced cancer [4].

The phase 3, multicenter, randomized, double-blind, placebo-controlled FRESCO study conducted in China demonstrated that fruquintinib significantly improved overall survival (OS) in patients with mCRC in a third-line or later setting compared to placebo. The median OS was 9.30 vs. 6.57 months (hazard ratio [HR] = 0.65, p < 0.001), and median progression-free survival (PFS) was 3.71 vs. 1.84 months (HR = 0.26, p < 0.001) [5]. Based on these results, fruquintinib was approved in China in September 2018 at a dose of 5 mg QD 3/1 on a 28 day cycle for the treatment of mCRC in patients who had failed 2 prior lines of systemic therapy [6].

The primary objective of the dose escalation phase of this phase 1/1b study was to evaluate the safety and tolerability of fruquintinib at 2 dose levels, 3 and 5 mg, orally (PO) QD, 3/1, and to confirm the RP2D in United States (U.S.) patients with advanced solid tumors. Secondary objectives were to evaluate the pharmacokinetics (PK) and preliminary anticancer activity of fruquintinib. In the dose expansion phase of this study, the primary objective was to assess fruquintinib anticancer activity at the RP2D. Here we report the dose escalation phase of the study, including the 3 and 5 mg dose cohorts, and the dose expansion cohort in patients with solid tumors of any type treated with the confirmed 5 mg dose of fruquintinib (Cohort A). The other 5 mg dose cohorts in the dose expansion phase included specific cancer types such as refractory mCRC and metastatic breast cancer and will be reported separately.

## Materials and methods

### Study design and patient selection

This was an open-label, phase 1/1b study of fruquintinib that enrolled U.S. patients with advanced solid tumors of any type (except squamous cell non-small cell lung cancer) conducted at 9 study centers in the U.S. In the dose escalation phase, eligible patients were adults ≥ 18 years old with histologically or cytologically confirmed, locally advanced or metastatic solid tumors who had progressed on approved systemic therapy and for which no effective treatment or standard of care was available. Additional key inclusion criteria included body weight ≥ 40 kg; at least 1 measurable target lesion according to Response Evaluation Criteria in Solid Tumors (RECIST) v1.1 per investigator assessment; Eastern Cooperative Oncology Group (ECOG) performance status of 0 or 1; and adequate hematologic, hepatic, and renal function. Key exclusion criteria included prior use of a VEGF inhibitor; systemic antineoplastic therapy or any investigational therapy within 4 weeks or 5 half-lives (whichever was shorter) of the first dose of study drug; uncontrolled hypertension; history of recent bleeding; history of gastrointestinal perforation or fistula; active cardiovascular disease; or recent thromboembolic events.

This study was conducted in accordance with the protocol, the ethical principles derived from international guidelines including the Declaration of Helsinki and Council for International Organizations of Medical Sciences International Ethical Guidelines, applicable International Council for Harmonisation of Technical Requirements for Pharmaceuticals of Human Use (ICH) Good Clinical Practice (GCP) and other guidelines, and applicable laws and regulations. Informed consent was obtained before the patient was entered into the study.

### Dose escalation and dose expansion

In the dose escalation phase, fruquintinib was administered sequentially in 2 successive cohorts, 3 and 5 mg PO QD on a 3/1 schedule on a 28-day cycle in a standard 3 + 3 design until the criteria for the MTD/RP2D were met. Dose-limiting toxicity (DLT)-evaluable patients must have met the following criteria: no previous anticancer therapy prior to DLT; completion of the first 28-day cycle with complete safety evaluation and reception of at least 85% of the assigned fruquintinib dose; or had a confirmed DLT during the first 28-day treatment cycle. If no more than 1 DLT occurred in the DLT observation window (Days 1–28 in Cycle 1 [C1]) among the 6 patients who received the 3 mg dose, DLT-evaluable patients were to be enrolled at the 5 mg dose level. If ≤ 1 patient experienced a DLT at the 5 mg dose level, then that dose level was declared the MTD/RP2D, and an additional 6 patients with solid tumors of any type were enrolled at the RP2D in the dose expansion phase (Cohort A). DLT assessment was only conducted for patients in the dose escalation phase.

A DLT was defined as any grade 4 non-hematologic adverse event (AE); any grade 3 non-hematologic AE related to study drug with the exception of nausea/vomiting, diarrhea, constipation, hypertension, and electrolyte imbalances downgraded within 3 days with appropriate supportive treatment; grade 4 neutropenia lasting > 3 days; grade 3 febrile neutropenia; grade 4 thrombocytopenia or grade 3 thrombocytopenia associated with bleeding; or dose interruption for > 14 days due to toxicity. The MTD was defined as the highest dose at which no more than 1 of 6 patients experienced a DLT.

Upon completion of dose escalation at the 3 and 5 mg dose levels and confirmation of the RP2D based on aggregated safety and PK data, additional patients were recruited in the expansion phase (Cohort A) at the RP2D to further characterize safety, tolerability, PK, and signals of clinical activity in patients with refractory solid tumors. Patients continued treatment until disease progression or any unacceptable toxicity, investigator decision to terminate therapy, or consent withdrawal.

### Safety assessments

Safety was assessed by evaluation of AEs, serious AEs (SAEs), AEs of special interest (AESIs), physical examinations, vital signs, single 12-lead electrocardiograms (ECGs) and cardiac monitoring, clinical laboratory data, and ECOG performance. AEs were graded using the National Cancer Institute Common Terminology Criteria for Adverse Events (CTCAE) v4.03. Safety was assessed from the date of first study drug administration to 37 days after the date of last study drug administration.

### Pharmacokinetic evaluations

Blood samples for determination of fruquintinib plasma concentrations were collected at pre-dose and 1, 2, 4, 8, and 24 h after dosing on Days 1, 14, and 21 of C1. The 24-h samples were collected prior to the next dose. PK samples were analyzed by LabCorp Bioanalytical (Shanghai, China) using a validated, specific, and sensitive liquid chromatography with tandem mass spectrometry assay method with an analytical range of 1.00 ng/mL (lower limit of quantitation) to 750 ng/mL (upper limit of quantitation). Inter-run variability was ≤ 5.2%. Quality control (QC) samples at 5 concentrations (3, 30, 45, 300, and 600 ng/mL) were assayed along with study samples, and the calculated QC sample concentrations deviated by ± 3.3% from the nominal concentrations.

PK parameters were determined by noncompartmental analysis using Phoenix® WinNonlin® v8.2. Systemic exposure to fruquintinib was evaluated based on PK parameters including maximum observed concentration (C_max_), time to C_max_ (T_max_), and area under the concentration curve from time 0 to 24 h post-dose (AUC_0-24_). Accumulation ratios (AR) for C_max_ and AUC were estimated from individual data and calculated as Day 14/Day 1 or Day 21/Day 1. The 3 mg dose cohort C_max_ and AUC_0-24_ values were dose-normalized to 5 mg for inclusion in box plots. Weight-normalized apparent clearance after oral administration (CL/F) was calculated as dose/AUC_0-24_ divided by body weight.

### Efficacy assessments

Tumor response was assessed according to RECIST v1.1 after every cycle (approximately 4 weeks) for the first 3 cycles and then every 8 weeks (± 7 days) thereafter. Assessments included computed tomography (CT) scans with oral or intravenous contrast (unless contraindicated) of the chest, abdomen, and pelvis or magnetic resonance imaging scans if CT contrast was contraindicated. Other assessment methods were used if clinically indicated. The assessment method used for each patient at baseline was the same throughout the study.

Best overall response (BOR) was determined using all time point responses (TPRs) up until the last evaluable TPR prior to or on the date of (i) radiological disease progression as defined by RECIST v1.1 or death; or (ii) loss to follow-up or withdrawal of consent; or (iii) receipt of subsequent anti-cancer medications, whichever was earlier. Investigator-assessed objective response rate (ORR) was defined as the proportion of patients with BOR of confirmed complete response (CR) or confirmed partial response (PR) according to RECIST v1.1. The interval for confirmation of CR and PR was at least 4 weeks. Disease control rate (DCR) was defined as the proportion of patients with a BOR of confirmed CR, confirmed PR, or stable disease (SD) lasting for at least 7 weeks. Duration of response (DoR) was calculated as the time (in months) from the date of first objective response (CR or PR confirmed after ≥ 4 weeks) until the date of the documented progression or death, whichever came first. PFS was defined as the time (months) from the date of first administration of study drug until the date of radiological disease progression or death due to any cause, whichever came first.

### Statistical analysis

The safety analysis set consisted of patients who received at least 1 dose of fruquintinib and was used in safety and PFS analyses. The efficacy analysis set included patients who received at least 1 dose of fruquintinib, had measurable target lesions at baseline, and had either at least 1 tumor assessment after treatment, or no tumor assessment after treatment but clinical progression as noted by the Investigator, or death due to disease progression before their first tumor scan after treatment. ORR and DCR were calculated with 2-sided 95% confidence intervals (CIs) using the Clopper-Pearson method [7]. DoR and PFS were estimated using the Kaplan–Meier method [8]. DoR was analyzed for patients with confirmed CR or PR. All statistical analyses were performed using SAS® v9.4 or higher (SAS Institute, Cary, NC).

## Results

### Patients

A total of 46 patients were screened for the dose escalation and dose expansion phases of the study, and 20 patients were enrolled in the study between 11 December 2017 and 05 March 2019 (Supplementary Fig. S1). In the dose escalation phase of the study, 7 patients were enrolled in the 3 mg dose cohort and 7 patients were enrolled in the 5 mg dose cohort. An additional 6 patients were then enrolled at the RP2D dose of fruquintinib 5 mg in the dose expansion phase (Cohort A). Results reported herein are based on the data cutoff of 14 January 2022. The safety analysis set included 20 patients with a median follow-up time of 12 months (95% CI: 3.9–26.2). The efficacy analysis set included 19 patients: 6 in the 3 mg dose cohort and 13 in the 5 mg dose cohort. One patient in the 3 mg dose cohort was excluded due to no post-baseline tumor assessment. All patients had discontinued treatment, most commonly for disease progression (65%), and no patients were still on the study.

Demographic and baseline characteristics for each cohort are shown in Table 1. Overall, the median age was 63 years (range: 36–77) with 55% < 65 years. Most patients were female (65%) and Caucasian (95%) with an ECOG score of 1 (85%). The primary tumor site in enrolled patients was breast in 5 (25%); colon in 4 (20%); ovary, head and neck, and pancreas each in 2 (10%); and pelvis, uterus, gall bladder, prostate, and small bowel each in 1 (5%). All patients had received at least 1 prior line of therapy, and 10 (50%) patients had received > 3 prior lines of therapy.

**Table 1:** Demographic and Baseline Characteristics of Patients

**Characteristic**	**3 mg Dose Cohort** **N = 7**	**5 mg Dose Cohort**^**a**^ **N = 13**	**Total** **N = 20**
**Age, years**			
Median (min, max)	61.0 (53.2, 69.4)	65.2 (35.6, 77.3)	63.2 (35.6, 77.3)
< 65, n (%)	5 (71.4)	6 (46.2)	11 (55.0)
≥ 65, n (%)	2 (28.6)	7 (53.8)	9 (45.0)
**Gender, n (%)**			
Male	3 (42.9)	4 (30.8)	7 (35.0)
Female	4 (57.1)	9 (69.2)	13 (65.0)
**Weight, kg**			
Median (min, max)	91.2 (66.7, 103.9)	69.0 (50.4, 114.8)	77.6 (50.4, 114.8)
**BMI, kg/m**^**2**^			
Median (min, max)	33.1 (24.9, 40.6)	23.4 (18.2, 43.4)	27.8 (18.2, 43.4)
**Race, n (%)**			
Caucasian	7 (100.0)	12 (92.3)	19 (95.0)
Black or African American	0	1 (7.7)	1 (5.0)
**Ethnicity, n (%)**			
Hispanic or Latino	1 (14.3)	1 (7.7)	2 (10.0)
Not Hispanic or Latino	6 (85.7)	12 (92.3)	18 (90.0)
**ECOG, n (%)**			
0	1 (14.3)	2 (15.4)	3 (15.0)
1	6 (85.7)	11 (84.6)	17 (85.0)
**Primary tumor site, n (%)**			
Breast	1 (14.3)	4 (30.8)	5 (25.0)
Colon	2 (28.6)	2 (15.4)	4 (20.0)
Ovarian	1 (14.3)	1 (7.7)	2 (10.0)
Head and neck	1 (14.3)	1 (7.7)	2 (10.0)
Pancreatic	1 (14.3)	1 (7.7)	2 (10.0)
Pelvic	1 (14.3)	0	1 (5.0)
Uterine	0	1 (7.7)	1 (5.0)
Gall bladder	0	1 (7.7)	1 (5.0)
Prostate	0	1 (7.7)	1 (5.0)
Small bowel	0	1 (7.7)	1 (5.0)
**Prior lines of therapy**^**b**^**, n (%)**			
1	1 (14.3)	2 (15.4)^c^	3 (15.0)^c^
2	2 (28.6)	3 (23.1)	5 (25.0)
3	1 (14.3)	1 (7.7)	2 (10.0)
> 3	3 (42.9)	7 (53.8)	10 (50.0)

*BMI* body mass index, *ECOG* Eastern Cooperative Oncology Group

^a^The 5 mg dose cohort is composed of the 7 patients enrolled in the dose escalation cohort and 6 patients enrolled in dose expansion Cohort A

^b^At least 5 prior anticancer therapies were received by 45% of patients. (Three patients had 8 or more prior lines.)

^c^A 77-year old white male patient with gallbladder cancer from Cohort A reported an unknown prior anticancer medication. Tumor metastasis was observed for this patient, and the disease stage at enrollment was IV. The patient lived 5.5 months after enrolling in the study, the primary reason of death was progressive disease, and the death was 2 days after the last dose

### Study drug exposure

The median duration of exposure for the 3 mg dose cohort was 3.7 months (range: 0.5–28.1) with a median of 4.0 cycles (range: 1–31). The median duration of exposure for the 5 mg dose cohort was 3.5 months (range: 0.6–11.3) with a median of 4.0 cycles (range: 1–11). Six patients, 3 (42.9%) in the 3 mg dose cohort and 3 (23.1%) in the 5 mg dose cohort, received > 6 cycles of treatment with fruquintinib. The median percentage-intended dose (PID) (defined as 100% times the duration [days] of receiving any dose of fruquintinib divided by the total duration [days] of exposure, calculated as last dose date of study drug – first dose date of study drug + 8 days) in the 3 mg dose cohort was 71.9% (range: 50.0%-74.3%). Given the 3/1 dosing schedule on a 28-day cycle, the reference value of PID is 75%. The median PID in the 5 mg dose cohort was 65.5% (range: 21.1%-75.9%). Four (57.1%) and 9 (69.2%) patients experienced dose interruption for the 3 and 5 mg dose cohorts, respectively.

### Safety

One (16.7%) of the 6 DLT evaluable patients from the 3 mg dose cohort who had a prior history of hypertension experienced 1 DLT of grade 4 hypertension that was managed with antihypertensive medication and resolved within 3 days. In addition, 1 patient in the 3 mg dose cohort was not DLT evaluable due to death from progressive disease (PD) 17 days after receiving study drug. No DLTs were reported in the 7 DLT-evaluable patients from the 5 mg dose cohort enrolled in dose escalation. With only 1 DLT reported in 1 cohort (3 mg), the MTD for fruquintinib was not reached. Safety data from the 3 and 5 mg dose escalation cohorts supported opening dose expansion enrollment. Based on the similarity of the safety and PK data between the U.S. and Chinese populations, the RP2D in the U.S. population was established and confirmed at 5 mg, which was consistent with the RP2D in the Chinese population.

All 20 patients experienced a treatment-emergent adverse event (TEAE) (Table 2). Five (71.4%) patients in the 3 mg dose cohort and 12 (92.3%) patients in the 5 mg dose cohort experienced a TEAE of grade ≥ 3. The most common AEs in the 3 mg dose cohort were constipation (5 [71.4%] patients); nausea and vomiting (4 [57.1%] patients each); and diarrhea, hypertension, abdominal pain, and abdominal pain upper (3 [42.9%] patients each).The most common AEs (by preferred term [PT]) in the 5 mg dose cohort were diarrhea, dysphonia, and hypertension (8 [61.5%] patients each); vomiting (7 [53.8%] patients); and constipation, decreased appetite, headache, and proteinuria (6 [46.2%] patients each).

**Table 2:** Incidence of Treatment-Emergent Adverse Events That Occurred in ≥ 10% of Patients

	**3 mg Dose Cohort**^**a**^ **N = 7, n (%)**		**5 mg Dose Cohort**^**a,b**^ **N = 13, n (%)**	
	**All grades**	**Grade ≥ 3**	**All grades**	**Grade ≥ 3**
Patients with any TEAE	7 (100)	5 (71.4)	13 (100)	12 (92.3)
**TEAEs by preferred term**				
Diarrhea	3 (42.9)	0	8 (61.5)	0
Dysphonia	2 (28.6)	0	8 (61.5)	0
Hypertension	3 (42.9)	2 (28.6)	8 (61.5)	5 (38.5)
Vomiting	4 (57.1)	0	7 (53.8)	0
Constipation	5 (71.4)	0	6 (46.2)	0
Decreased appetite	2 (28.6)	0	6 (46.2)	0
Headache	1 (14.3)	0	6 (46.2)	0
Proteinuria	2 (28.6)	0	6 (46.2)	0
Fatigue	1 (14.3)	0	5 (38.5)	2 (15.4)
Nausea	4 (57.1)	0	5 (38.5)	0
Cough	2 (28.6)	0	4 (30.8)	1 (7.7)
Dehydration	1 (14.3)	0	4 (30.8)	0
Dizziness	2 (28.6)	0	4 (30.8)	0
Dyspepsia	2 (28.6)	0	4 (30.8)	0
Hematuria	0	0	4 (30.8)	1 (7.7)
Oral dysesthesia	0	0	4 (30.8)	0
Weight decreased	2 (28.6)	0	4 (30.8)	0
Abdominal pain	3 (42.9)	0	3 (23.1)	1 (7.7)
Arthralgia	1 (14.3)	0	3 (23.1)	0
Blood ALP increased	0	0	3 (23.1)	1 (7.7)
Flatulence	0	0	3 (23.1)	0
Hypothyroidism	1 (14.3)	0	3 (23.1)	0
Stomatitis	2 (28.6)	0	3 (23.1)	0
Upper respiratory tract infection	2 (28.6)	0	3 (23.1)	1 (7.7)
Anxiety	2 (28.6)	0	2 (15.4)	0
Back pain	0	0	2 (15.4)	0
Dyspnea	1 (14.3)	1 (14.3)	2 (15.4)	1 (7.7)
Muscular weakness	1 (14.3)	0	2 (15.4)	0
Oral pain	0	0	2 (15.4)	1 (7.7)
Pain in extremity	0	0	2 (15.4)	0
PPE syndrome	0	0	2 (15.4)	1 (7.7)
Rash papular	0	0	2 (15.4)	0
Upper abdominal pain	3 (42.9)	0	2 (15.4)	0
Vascular access complication	1 (14.3)	0	2 (15.4)	0
Hypokalemia	1 (14.3)	1 (14.3)	1 (7.7)	1 (7.7)
Myalgia	1 (14.3)	0	1 (7.7)	0
Pyrexia	1 (14.3)	0	1 (7.7)	0
Hyperglycemia	1 (14.3)	0	0	0
INR increased	1 (14.3)	0	0	0
Urinary tract infection	1 (14.3)	0	0	0

*ALP* alkaline phosphatase, *DLT*, dose limiting toxicity; *INR* international normalized ratio, *PPE* palmar-plantar erythrodysesthesia, *TEAE* treatment-emergent adverse event

^a^One patient in the 3 mg dose escalation cohort experienced a DLT of grade 4 hypertension, and none of the 7 patients in the 5 mg cohort of the dose escalation phase experienced a DLT

^b^The 5 mg dose cohort is composed of the 7 patients enrolled in the dose escalation cohort and 6 patients enrolled in dose expansion Cohort A

TEAEs leading to dose interruption, dose reduction, and dose discontinuation occurred in both dose cohorts. Four (57.1%) patients in the 3 mg dose cohort and 8 (61.5%) patients in the 5 mg dose cohort experienced a TEAE leading to dose interruption. Two (28.6%) patients in the 3 mg dose cohort and 4 (30.8%) patients in the 5 mg dose cohort experienced a TEAE leading to dose reduction. One (14.3%) patient in the 3 mg dose cohort and 3 (23.1%) patients in the 5 mg dose cohort experienced a TEAE that led to treatment discontinuation. Three (42.9%) patients in the 3 mg cohort and 5 (38.5%) patients in the 5 mg dose cohort died from PD.

### Pharmacokinetics

**Fig. 1:**
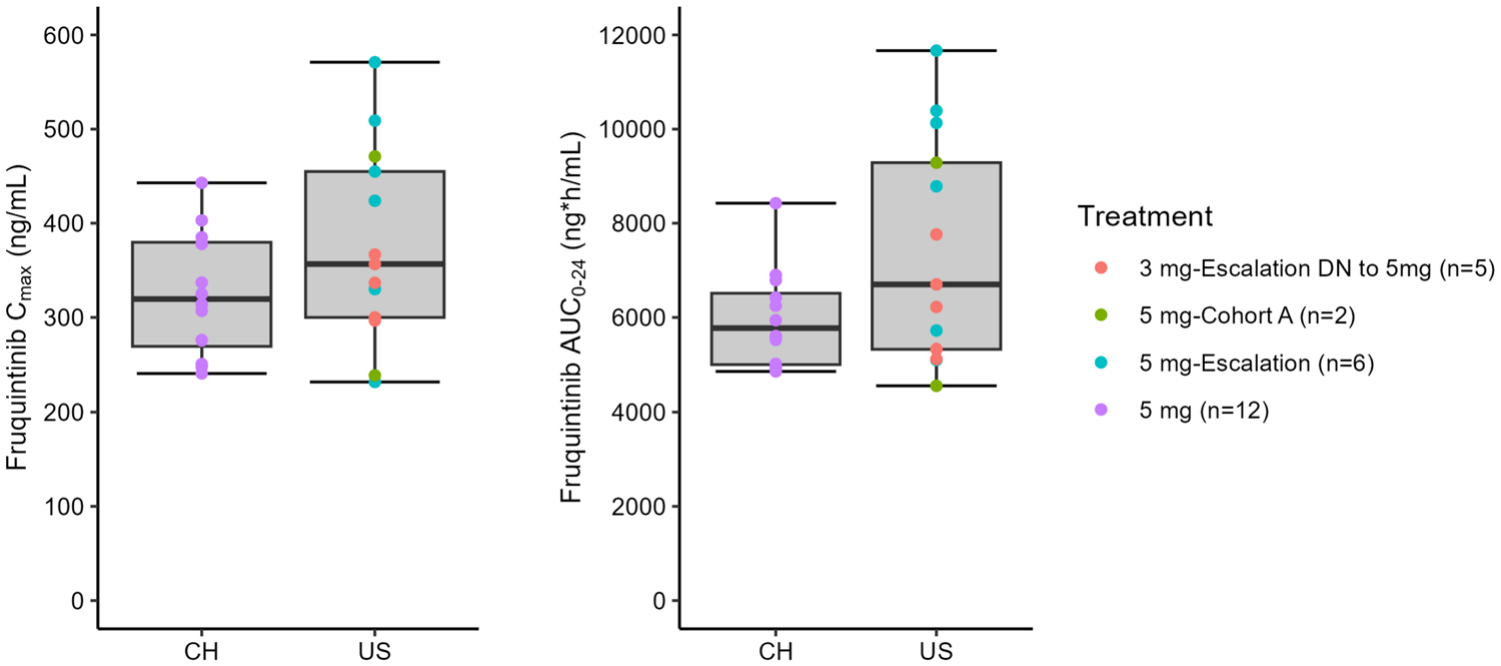
Comparison of Fruquintinib Steady-State Pharmacokinetic Exposures Across Studies. CH: Includes C1D21 data from Chinese patients in Study 2012–013-00CH3, NCT01975077, a phase 1b, randomized open-label study in patients with advanced CRC that had failed ≥ 2 lines of prior therapy in the 5 mg 3/1 dose cohort. US: Includes C1D14 data from US patients in the study reported in this manuscript (Study 2015–013-00US1; NCT03251378). PK parameters from the 3 mg dose cohort were dose-normalized to 5 mg. The median line is shown. The lower and upper hinges correspond to the first and third quartiles (i.e., the 25th and 75th percentiles, respectively). The upper whisker extends from the hinge to the largest value no further than 1.5 * IQR from the hinge (where IQR is the inter-quartile range or distance between the first and third quartiles). The lower whisker extends from the hinge to the smallest value at most 1.5 * IQR of the hinge. Data beyond the end of the whiskers are outliers. *3/1* 3 weeks on and 1 week off, *AUC*_*0-24*_ area under the concentration curve from time 0 to 24 h post-dose, *C* Cycle, *CH* China, *C*_*max*_ maximum observed concentration, *D* Day, *DN* dose-normalized, *IQR* interquartile range, *PK* pharmacokinetics, *US* United States

The PK of fruquintinib was evaluated in 20 patients: 7 patients in the dose escalation phase at 3 mg QD and 13 patients (7 in dose escalation and 6 patients in dose expansion) at 5mg QD. PK was evaluated in C1 after a single dose (Day 1) and at steady-state (Days 14 and 21) as summarized in Table 3. Evaluable data in dose expansion Cohort A was limited with AUC_0-24_ from 3, 2, and 1 patient(s) on Days 1, 14, and 21, respectively. Fruquintinib was quickly absorbed with measurable plasma concentrations within 1 h after oral administration (Supplementary Fig. S2). Median T_max_ occurred approximately 2 h after administration (Table 3). Plasma trough concentrations of fruquintinib increased steadily with time and in a dose-related manner. The mean weight-normalized CL/F was comparable between the 3 and 5 mg dose groups (0.17 mL/min/kg on Day 14), suggesting that fruquintinib PK was linear and dose independent. Systemic exposure (C_max_ and AUC_0-24_) was comparable on Days 14 and 21, indicating that fruquintinib reached steady-state exposure after 14 days of daily administration. The time to reach steady-state (within 14 days) is consistent with the fruquintinib elimination half-life of 42 h reported in the previous study [3]. Systemic exposure to fruquintinib was approximately four-fold higher at steady-state compared to the first dose. The systemic exposure parameters between the current study in U.S. patients and the previous study in Chinese patients are generally similar and overlap (Fig. 1).

**Table 3:** Comparison of Pharmacokinetic Profile for Fruquintinib in U.S. Patients

**Study Population**	**Parameter**	**Escalation Phase**		**Expansion Phase**	**Total**^**a**^
		**3 mg**	**5 mg**	**Cohort A (5 mg)**	**5 mg**
**Cycle 1 Day 1**		**N = 7**	**N = 7**	**N = 6**	**N = 13**
	C_max_ (ng/mL), GM (%CV)	52.2 (24.2)	114 (27.5)	96.0 (23.0)	105 (26.1)
	T_max_ (h), median (min–max)	2.07 (1.03–21.2)	1.95 (0.92–25.0)	1.96 (1.00–13.8)	1.95 (0.92–25.0)
	AUC_0-24_ (ng•h/mL), GM (%CV)	912 (21.5)^b^	2010 (19.2)	1340 (25.6)^**c**^	1780 (28.2)^d^
**Cycle 1 Day 14**		**N = 5**	**N = 6**	**N = 2**	**N = 8**
	C_max_ (ng/mL), GM (%CV)	198 (9.8)	403 (33.7)	336	385 (35.2)
	T_max_ (h), median (min–max)	1.03 (0.97–1.92)	2.00 (1.97–7.05)	1.52 (1.00–2.03)	2.00 (1.00–7.05)
	AUC_0-24_ (ng•h/mL), GM (%CV)	3694 (17.1)	8249 (35.2)	6507	7774 (37.5)
	AR AUC_0-24_, mean (SD)	4.24 (1.21)	4.19 (1.19)	4.04^e^	4.17 (1.09)^f^
	AR C_max_, mean (SD)	4.06 (1.42)	3.66 (1.42)	3.57	3.64 (1.21)
	Weight Normalized CL/F (mL/min/kg), mean (SD)	0.17 (0.05)^g^	0.18 (0.06)	0.14	0.17 (0.06)
**Cycle 1 Day 21**		**N = 5**	**N = 6**	**N = 1**	**N = 7**
	C_max_ (ng/mL), GM (%CV)	205 (12.8)	390 (19.1)	238	364 (25.9)
	T_max_ (h), median (min–max)	1.83 (0.92–1.98)	2.00 (1.88–4.08)	1.00	2.00 (1.00–4.08)
	AUC_0-24_ (ng•h/mL), GM (%CV)	3731 (22.5)	7740 (26.7)	4995	7271 (29.7)
	AR AUC_0-24_, mean (SD)	4.36 (1.53)	3.90 (0.96)	4.43	3.98 (0.902)
	AR C_max_, mean (SD)	4.22 (1.45)	3.47 (1.22)	3.40	3.46 (1.12)
	Weight Normalized CL/F (mL/min/kg), mean (SD)	0.15 (0.05)^g^	0.18 (0.06)	0.15	0.18 (0.06)

*AR* accumulation ratio, *AUC*_*0-24*_ area under the concentration curve from time 0 to 24 h post-dose, *CL/F* apparent clearance, *C*_*max*_ maximum observed concentration, *CV* coefficient of variation, *GM* geometric mean, *SD* standard deviation, *T*_*max*_ time to C_max_, *U.S.* United States

^a^The total cohort is composed of the 7 patients enrolled in the dose escalation cohort at the 5 mg dose and 6 patients enrolled in dose expansion Cohort A

Note: Individual data is presented when n = 1 and geometric mean or mean (without %CV or SD) is presented when n = 2

^b^n = 6; ^c^n = 3; ^d^n = 10; ^e^n = 1; ^f^n = 7; ^g^n=4

### Clinical activity

**Fig. 2:**
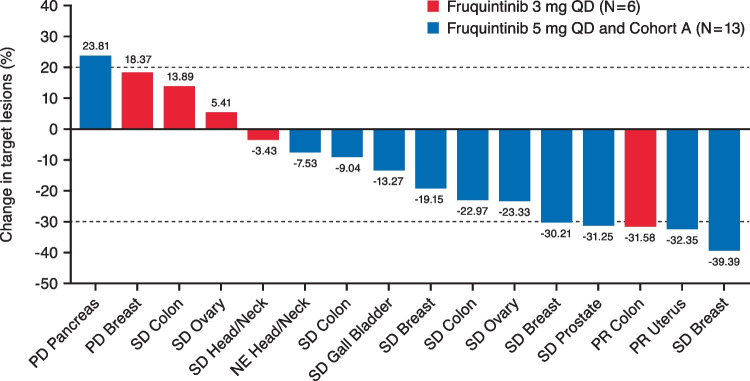
Tumor Burden Change for Sum of Target Lesions Diameters (mm): Best Relative Change from Baseline (%). The best relative change from baseline (%) in tumor burden for the sum of target lesion diameters (mm) is shown for the 3 mg (red bars) and 5 mg (blue bars) dose cohorts, respectively. Sixteen patients were evaluable; 1 patient from the 3 mg dose cohort and 2 patients from the 5 mg dose cohort did not have post-baseline tumor scans before death. Notes: Data are presented for all patients with at least 1 post-baseline tumor measurement. Dashed lines indicate cutoff for PR (-30%) and PD (+ 20%). The label of x-axis tick is a combination of the best overall response of patient and the primary malignancy site. The 5 mg dose cohort is composed of 7 patients enrolled in the dose escalation cohort at the 5 mg dose and 6 patients enrolled in the dose expansion Cohort A. *NE* not evaluable, *PD* progressive disease, *PR* partial response, *QD* once daily, *SD* stable disease

Tumor response according to investigator assessment by RECIST v1.1 was evaluated in 19 of the 20 treated patients in the study (Supplementary Table S1); 1 patient in the 3 mg dose cohort was not evaluable because of premature withdrawal due to an AE that prevented post-baseline tumor assessment. A waterfall plot of the best percentage change from baseline in sum of diameters of target lesions is presented in Fig. 2. No patient in either dose cohort had a CR. In the 3 mg cohort, 1 patient with metastatic adenocarcinoma of rectal origin had a confirmed PR for an ORR of 16.7% (95% CI: 0.42–64.12); 3 (50%) patients had a BOR of SD for a DCR of 66.7% (95% CI: 22.28–95.67). In the 5 mg cohort, 1 patient with endometrial adenocarcinoma had a confirmed PR for an ORR of 7.7% (95% CI: 0.19–36.03); 8 (61.5%) patients had a BOR of SD for a DCR of 69.2% (95% CI: 38.57–90.91).

Of 2 patients with a confirmed PR, the first patient was a 64-year-old white male with metastatic adenocarcinoma of rectal origin who had received prior anticancer treatment with oxaliplatin, fluorouracil, bevacizumab, irinotecan, folinic acid, bintrafusp alfa, an investigational drug, and radiation. He was started on the 3 mg dose with a PR after C4 (confirmed after C6). His fruquintinib dose was increased to the RP2D of 5 mg during C13 given the observed benefit at the discretion of the investigator, and with sponsor approval the patient received fruquintinib for another 18 cycles. During this extended treatment period, SD was maintained, and a dose reduction to 4 mg occurred due to oral mucositis in C28. He discontinued study drug at C31 due to PD (i.e., 28.6 months after the first study drug administration). His DoR was 7.6 months The second patient was a 69-year-old white female with endometrial adenocarcinoma who received prior treatment with paclitaxel, carboplatin, docetaxel, and radiation. She received fruquintinib 5 mg for 8 cycles. She discontinued study drug due to an embolic stroke that the investigator attributed to fruquintinib in C8. She demonstrated a PR after C4 (confirmed after C6 and C7), and her response was ongoing when she started gemcitabine treatment 3.7 months after the first assessment of PR.

Median follow up was 18.0 months (range: 0.6–28.7) in the 3 mg cohort and 11.1 months (range: 1.9–26.2) in the 5 mg dose cohort. In the 3 mg dose cohort, 6 (85.7%) patients had PFS events, and the median PFS was 6.90 months (95% CI: 0.56, not estimable) (Supplementary Fig. S3). In the 5 mg dose cohort, 5 (38.5%) patients had PFS events, and the median PFS was 5.49 months (95% CI: 1.77, not estimable).

## Discussion

This open-label, phase 1/1b study was conducted to evaluate the safety, tolerability, PK characteristics, and preliminary anticancer activity at the RP2D of fruquintinib in U.S. patients to confirm the RP2D established in China. Fruquintinib was well tolerated in both the 3 and 5 mg dose cohorts. One DLT (grade 4 hypertension) was observed in the 3 mg dose cohort, and no DLTs were observed in the 5 mg dose cohort. The RP2D was established as 5 mg PO, 3/1, on a 28-day cycle. The majority of TEAEs at the RP2D were grade 1 or 2. The overall safety profile was consistent across patient dose cohorts and tumor types and consistent with previous fruquintinib studies.

The PK profile of fruquintinib in this study is comparable to the PK profile established in studies in China. The dose-normalized exposure parameters between studies in U.S. patients and studies in Chinese patients were generally similar and overlapping, suggesting that the country of origin has no meaningful impact on fruquintinib PK.

Anticancer activity was observed in this heavily pretreated population. In the fruquintinib 3 mg dose cohort, the ORR was 16.7% with a DCR of 66.7% and estimated median PFS of 6.9 months. In the fruquintinib 5 mg dose cohort, the ORR was 7.7% with a DCR of 69.2% and estimated median PFS of 5.49 months.

## Conclusion

Fruquintinib was generally well tolerated in these patients, with a safety profile consistent with that of other studies conducted in China. The RP2D in U.S. patients is the same as the approved dose in China, and PK characteristics are consistent with those in the dose-finding study conducted in China. Preliminary anticancer activity was evident in these patients with advanced solid tumors. Based on the results of this phase 1/1b study and earlier studies, further investigation of fruquintinib in patients with mCRC was conducted in a global phase 3 study along with a phase 1b/2 study of fruquintinib in combination with tislelizumab, an anti-PD-1 monoclonal antibody, in patients with solid tumors.

### Supplementary Information

Below is the link to the electronic supplementary material.

Supplementary file1 (PDF 374 KB)

## Data Availability

Data generated or analyzed during this study are included in this published article and its supplementary information files. Additional data/analyses can be provided upon request.
