# Supplementary material for: Phase 1/1b open-label, dose-escalation study of fruquintinib in patients with advanced solid tumors in the United States
Source: Invest New Drugs. 2023 Oct 5;41(6):851–60. doi: 10.1007/s10637-023-01395-y (PMC10663261; doi:10.1007/s10637-023-01395-y)
Supplement: Supplementary file 1 — Supplementary file1 (PDF 374 KB) [file 10637_2023_1395_MOESM1_ESM.pdf]

Supplementary Information

Phase 1/1b open-label, dose-escalation study of fruquintinib in patients with advanced solid tumors in the United States

Andrea Wang-Gillam, MD, PhD[1], William Schelman, MD, PhD[2], Stacey Ukrainskyj, BSN[2], Caly Chien, PhD[2], Martha Gonzalez, BS[2], Zhao Yang, PhD[2], Marek Kania, MD, MBA[2], and Heather Yeckes-Rodin, MD[3]

[1]Washington University School of Medicine, St. Louis, MO, USA; [2]HUTCHMED International Corporation, Florham Park, NJ, USA; [3]Hematology Oncology Associates of the Treasure Coast, Port St. Lucie, FL, USA

Contents

Figures 2

Supplementary Fig. S1 Patient Disposition 2

Supplementary Fig. S2 Mean Fruquintinib Concentration-Time Profiles on Days, 1, 14, and 21........ 4

Supplementary Fig. S3 Kaplan-Meier Curve for Progression-Free Survival 5

Tables 6

Supplementary Table S1 Summary of Anti-tumor Response by Dose Group........ 6

Figures

Supplementary Fig. S1 Patient Disposition

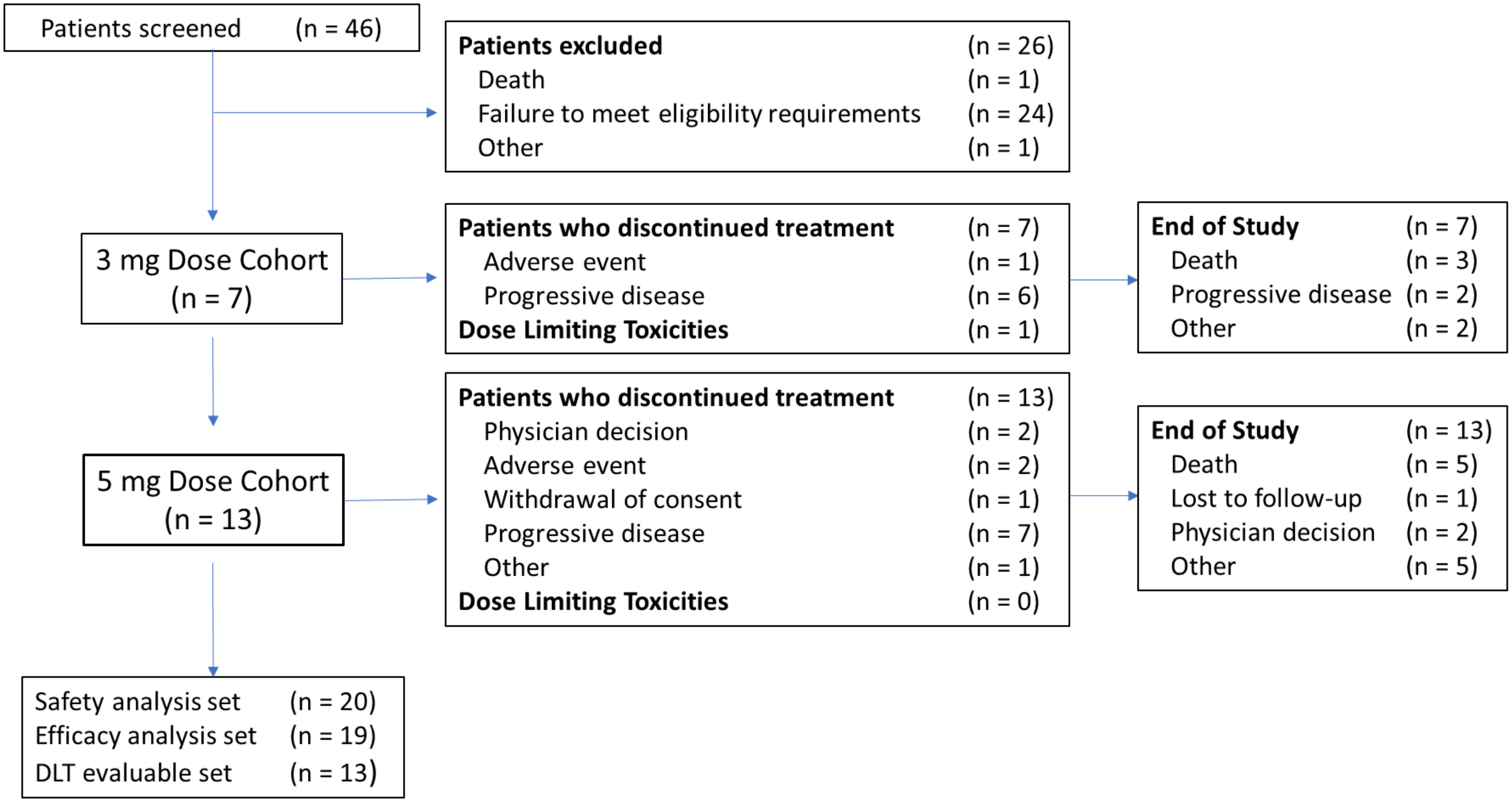

A total of 46 patients was screened for the dose escalation and dose expansion parts of the study, and 20 patients were enrolled in this Phase 1/1b study between 11 December 2017 and 05 March 2019 at 2 dose levels: 7 patients at the 3 mg dose and 13 patients at the 5 mg dose. Note: The 5 mg dose cohort is comprised of 7 patients enrolled in the dose escalation cohort and 6 patients enrolled in the dose expansion Cohort A. A 74-year old white female patient from Cohort A discontinued the study drug with primary reason of progressive disease instead of

adverse event though this patient had a reported drug-related grade 3 serious adverse event of dyspnea which led to study drug withdrawal. Of note, the outcome of the adverse event was recovered/resolved which occurred 1 week after the discontinuation of study drug.
DLT dose limiting toxicity

Supplementary Fig. S2 Mean Fruquintinib Concentration-Time Profiles on Days, 1, 14, and 21

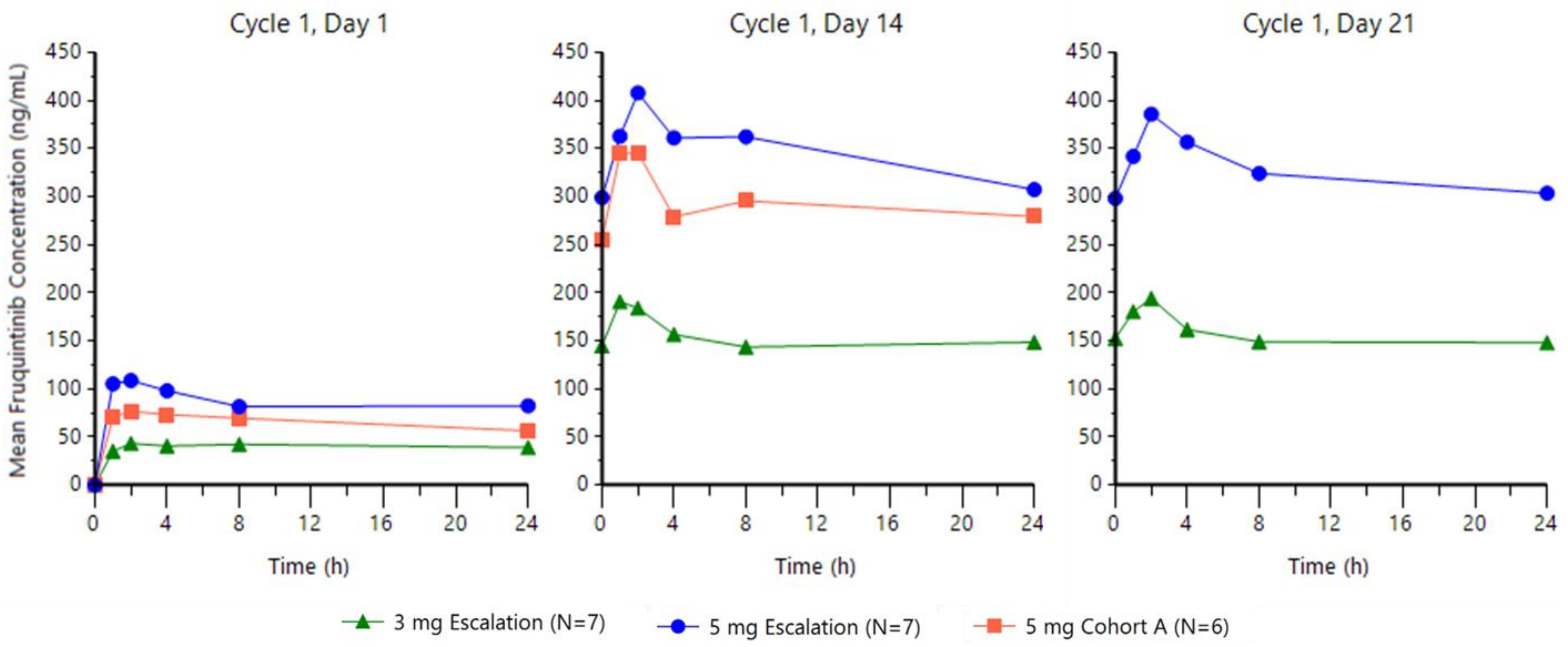

Note: For the PK data, the 5 mg dose cohort of 7 patients enrolled in dose escalation and 6 patients in dose expansion Cohort A are presented separately due to limited data availability. N=5 for 3 mg Escalation and N=6 for 5 mg Escalation on Day 14 and Day 21. Mean data for Cohort A on Day 21 were not available as N=1.

Supplementary Fig. S3 Kaplan-Meier Curve for Progression-Free Survival

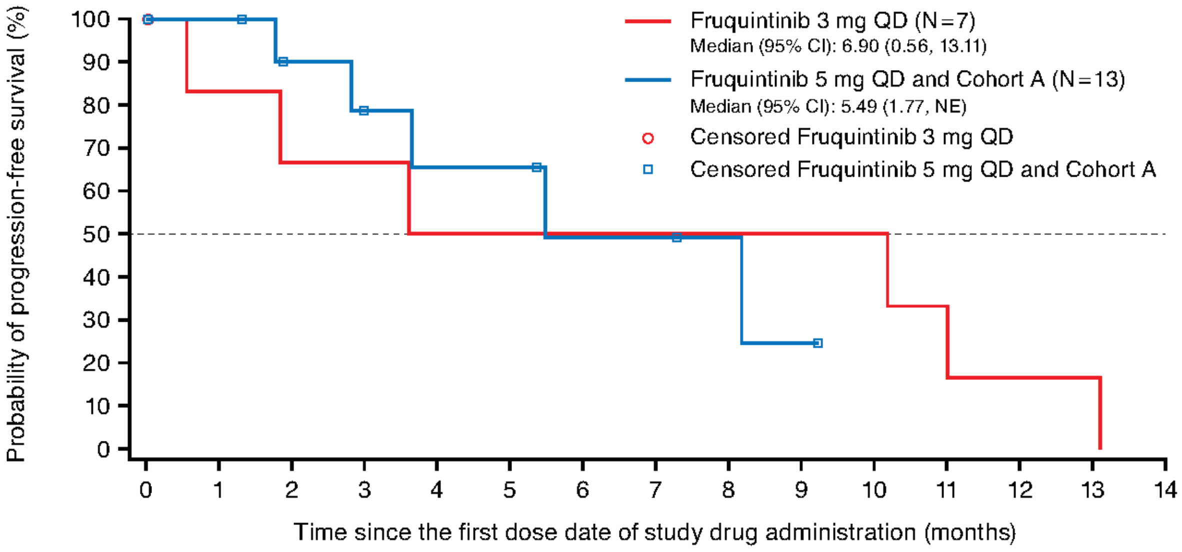

Number of patients at risk

	0	1	2	3	4	5	6	7	8	9	10	11	12	13	14
3 mg QD	7	5	4	4	3	3	3	3	3	3	3	2	1	1	0
5 mg QD and Cohort A	13	11	8	6	5	5	3	3	2	1	0				

Kaplan-Meier curves for progression free survival for the 3 mg (red line) and 5 mg (blue line) dose cohorts are shown with patients censored and number of patients at risk. Note: The 5 mg dose cohort is comprised of 7 patients enrolled in the dose escalation cohort and 6 patients in the dose expansion Cohort A.

CI confidence interval, *QD* once daily, *NE* not estimable

Tables

Supplementary Table S1. Summary of Anticancer Response by Dose Group

	**3 mg Dose Cohort (N = 6) n (%)**	**5 mg Dose Cohort[a] (N = 13) n (%)**
**Best overall response (confirmed)**		
CR	0	0
PR	1 (16.7)	1 (7.7)
SD	3 (50.0)	8 (61.5)
PD	1 (16.7)	1 (7.7)
Not evaluable	0	1 (7.7)
Not assessable [b]	1 (16.7)	2 (15.4)
ORR (CR+PR), n (%) [95% CI]	1 (16.7) [0.42, 64.12]	1 (7.7) [0.19, 36.03]
DCR (CR+PR+SD for 7 weeks), n (%) [95% CI]	4 (66.7) [22.28, 95.67]	9 (69.2) [38.57, 90.91]
ORR regardless of confirmation, n (%) [95% CI]	1 (16.7) [0.42, 64.12]	4 (30.8) [9.09, 61.43]

[a] The 5 mg dose cohort is comprised of 7 patients enrolled in the dose escalation cohort and 6 patients in the dose expansion Cohort A.
[b] Not assessable included patients who did not have baseline tumor assessment or did not have any post-baseline tumor assessment.
Note: The 95% CI was based on the Clopper-Pearson method.
CI confidence interval, *CR* complete response, *DCR* disease control rate, ORR objective response rate, *PD* progressive disease, *PR* partial response, *SD* stable disease
